# Introduction to ‘Artificial intelligence in failure analysis of transportation infrastructure and materials'

**DOI:** 10.1098/rsta.2022.0177

**Published:** 2023-09-04

**Authors:** Yue Hou, Qiao Dong, Dawei Wang, Jenny Liu

**Affiliations:** ^1^ Department of Civil Engineering, Swansea University, Swansea SA1 8EN, UK; ^2^ School of Transportation, Southeast University, Nanjing, Jiangsu Province 21189, People's Republic of China; ^3^ School of Transportation Science and Engineering, Harbin Institute of Technology, Harbin 150090, People's Republic of China; ^4^ RWTH Aachen University, Aachen 52074, Germany; ^5^ Department of Civil Architectural and Environmental Engineering, Missouri University of Science and Technology, MO 65409, USA

**Keywords:** transportation infrastructure, failure analysis, artificial intelligence

## Abstract

Transportation infrastructures, including roads, bridges, tunnels, stations, airports and subways, play fundamental roles in modern society. Engineering failures of transportation infrastructures may result in significant damage to the public. The traditional methods are to monitor, store and analyse the information during the infrastructure and material design, testing, construction, numerical simulations, evaluation, operation, maintenance and preservation, using mechanistic-based, material-based and statistics-based approaches. In recent decades, artificial intelligence (AI) has drawn the attention of many researchers and has been used as a powerful tool to understand and analyse the engineering failures in transportation infrastructure and materials. AI has the advantages of conveniently characterizing infrastructure materials in multi-scale, extracting failure information from images and cloud points, evaluating performance from the signals of sensors, predicting the long-term performance of infrastructure based on big data and optimizing infrastructure maintenance strategies, etc. In the future, AI techniques will be more effective and promising for data collection, transmission, fusion, mining and analysis, which will help engineers quickly detect, analyse and finally prevent the engineering failures of transportation infrastructure and materials. This theme issue presents the latest developments of AI in failure analysis of transportation infrastructure and materials.

This article is part of the theme issue 'Artificial intelligence in failure analysis of transportation infrastructure and materials'.

Transportation infrastructures, including roads, bridges, tunnels, stations, airports and subways, play fundamental roles in modern society. Engineering failures of transportation infrastructures may result in significant damage to the public. In recent decades, artificial intelligence (AI) has drawn the attention of many researchers and been used as a powerful tool to understand and analyse the engineering failures in transportation infrastructure and materials. This theme issue presents the latest developments of AI in failure analysis of transportation infrastructure and materials.

Road is one of the most commonly seen transportation infrastructures and many researchers have used AI to determine the distresses and failures, especially for road crack detection. Lv *et al*. [1] proposed automatic identification of pavement cracks in public roads using an optimized deep convolutional neural network model. Their study aimed to improve the efficiency of automatic identification of pavement distress and improve the status quo of difficult identification and detection of pavement distress. First, the identification method of pavement distress and the types of pavement distress were analysed. Then the design concept of deep learning in pavement distress recognition was described. Finally, the mask region-based convolutional neural network (Mask R-CNN) model was designed and applied in the recognition of road crack distress. The results show that in the evaluation of the model's comprehensive recognition performance, the highest accuracy was 99%, and the lowest accuracy was 95% after the test and evaluation of the designed model in different datasets. Lin *et al*. [2] also proposed an AI-based method to analyse road crack. In their study, an alternative to the sequence-to-sequence perspective with a transformer network termed TransCrack was introduced for road crack detection. Specifically, an image was decomposed into a grid of fixed-sized crack patches that was flattened with position embedding into a sequence. They further proposed a pure transformer-based encoder with multi-head reduced self-attention modules and feed-forward networks for explicitly modelling long-range dependencies from the sequential input in a global receptive field. Results showed that the proposed TransCrack achieves state-of-the-art performance over all counterparts by a substantial margin, and qualitative results further demonstrate its superiority in contiguous crack recognition and fine-grained profile extraction.

AI can assist the detection and analysis of road service conditions using advanced testing instruments. The falling weight deflectometer (FWD) test is a common non-destructive testing method for evaluating the structural capacity of pavements. At present, the data processing of the FWD tests mainly focuses on the deflection data, while paying less attention to the deflection-time history. Because FWD is equipped with impulse loads and geophones, which allow for the generation and capture of surface wave signals propagation, it was hypothesized that Rayleigh wave dispersion theory can be applied to calculate the modulus profile along the pavement depth by analysing the dispersive properties of deflection signal measured during FWD tests. To test the hypothesis, Xue Wang *et al*. [3] developed a new methodology for the FWD test and data analysis, referred to as the FWD dispersion curve method. They firstly introduced the concept of the new method, followed by illustrating the procedure and the experimental set-up. Case studies on three concrete pavement segments were then presented to evaluate the effectiveness of the FWD dispersion curve method. Modifications to the existing FWD device were further recommended on the impact loading sources and signal collection themes so that the modulus of a much shallower layer such as the concrete slab and upper asphalt layers can be obtained. Deng *et al*. [4] conducted a feasibility study of determining the asphalt pavement condition from test and finite element model updating. Their study used a finite element model and FWD test to determine the asphalt pavement condition by applying AI-based algorithms for automatic and intelligent determination. The major work included: (i) conducting a sensitivity analysis associating material properties with their most influential responses; (ii) applying various optimization algorithms to identify alternative solutions in the high-dimensional problem; and (iii) comparing different backcalculation schemes and settings.

AI can be directly used in road structure and material performance evaluation. Luo *et al*. [5] proposed a hybrid approach for fatigue life prediction of in-service asphalt pavement. They aimed to propose a hybrid approach to fill this gap. The key idea was that the damage condition was backcalculated by an AI-based finite element model updating using field monitoring information (data-driven component), which was used to update the parameters in a mechanistic composition-specific fatigue life prediction equation (model-driven component). The laboratory test of field cores gave the material non-destructive properties. The simulated pavement response subjected to truck loading showed a good agreement with measured values, which indicated that the verified constitutive relationship could be used in the data-driven component. Deng *et al*. [4] used a recurrent neural network considering maintenance to predict urban roads performance in Beijing. They developed a predictive model with the cost of different types of maintenance works data that reflected the continuous true usage performance of the pavement. The model was trained based on the dataset containing 5-year maintenance works on urban roads in Beijing with the pavement performance indicators for the corresponding years. The same roads were matched and combined to obtain a set of sequences of pavement performance changes with the features of the current year and, combined with the recurrent neural network-based long short-term memory network and gate recurrent unit network, the prediction accuracy of the highway pavement performance in the test set was significantly increased.

The AI-based evaluation of transportation materials performance can also serve as a reference for future functional roads. Dawei Wang *et al*. [6] evaluated the dielectric model of asphalt pavement materials towards the future electrified road (e-road). The dielectric properties of asphalt mixture are crucial for the future e-road and pavement non-destructive detection. To quantify the influence of temperature and frequency on the dielectric properties of asphalt mixtures, the dielectric constants, dielectric loss factor and dielectric loss tangents of aggregate, asphalt binders and asphalt mixtures were tested over the temperature range of −30 to 60°C and frequency range of 200 to 2 000 000 Hz. The results showed that the dielectric constants and dielectric loss factors of aggregate, asphalt binders and asphalt mixtures varied linearly with temperature, while the growth rates varied with the frequency. A model based on the nonlinear fitting was first presented to estimate the dielectric loss factor, and another prediction model of the dielectric constant of asphalt mixtures considering the temperature impact was proposed afterwards. Pan *et al*. [7] conducted an automatic pavement texture recognition using lightweight few-shot learning. Their study proposed a few-shot learning model based on a Siamese network for pavement texture recognition with a limited dataset. The model achieved 89.8% accuracy in a four-way five-shot task classifying the pavement textures of dense asphalt concrete, micro surface, open-graded friction course and stone matrix asphalt.

AI could also provide assistance in assessing the impacts of autonomous vehicles on pavement performance. Chen *et al*. [8] proposed an apple-to-apple comparison that was first performed to systematically reveal the behavioural differences between the human-driven vehicle (HDV) and CAV trajectory patterns for the first time, with the data collected from a camera-based NGSIM dataset and autonomous driving co-simulation platform, CARLA and SUMO, respectively. A gradient boosting-based ensemble learning model for pavement performance (i.e. International Roughness Index) prediction was then developed with the input features including three driving pattern features, namely, lateral wandering deviation, longitudinal car-following distance and driving speed, plus 20 other context variables. A total of 1707 observations were extracted from the long-term pavement performance database for model training purposes. The result indicated that the trained model can accurately predict pavement deterioration, and that CAV deteriorated pavement faster than HDV by 8.1% on average.

Monitoring and analysis of performance and safety issues of underground transportation infrastructures, like tunnel, are very important for transportation infrastructure engineers. It has been a hot spot for researchers using AI to solve the relevant problems. Li *et al*. [9] proposed an intelligent decision method for stability assessment of shield tunnel based on multi-objective data mining. Their research focused on the limit support pressure and the excavation face stability in the soil when crossing the Yangtze River. First, the analytical formula of limit support pressure of the excavation face was established through the wedge model. The support safety coefficient was given to assess the excavation face stability quantitatively. Then the rough set algorithm was used to analyse the sensitivity of each index to establish the reduced evaluation index system for the excavation face stability. The BP neural network was used to train the learning data, and a neural network evaluation model with a prediction error of 5.7675 × 10^–4^ was established. The prediction performance of BP was verified by comparing the TOPSIS prediction model and the cloud model. Jin *et al*. [10] conducted a machine learning-based identification of segment joint failure in underground tunnels. The traditional method of detecting dislocation or opening has the problem of high labour costs. Their study proposed an identification method of segment joint failure in underground tunnels based on the back-propagation neural network (BPNN) to overcome the problem. First, their study collected the tunnel settlement curves of different subways in the soft soil area of East China. It calculated tunnel settlement dislocation and settlement-opening datasets using the equivalent axial stiffness model. Then the corresponding BPNN regression model was established. Finally, the new settlement curve was input into the regression model to predict the dislocation and the opening to judge whether the segment joint wasinvalid.

Rail is a very important transportation infrastructure. Its corrugation is one of the key problems that significantly affects metro safety. Cai *et al*. [11] conducted machine learning-based rail corrugation recognition from a metro vehicle response and noise perspective. In their study, a particle probabilistic neural network algorithm (PPNN) was developed. The PPNN was incorporated with the particle swarm optimization algorithm and the probabilistic neural network (PNN). On the basis of the above, the in-vehicle noise characteristics measured in the field were used to recognize normal rail wavelengths of 30 and 50 mm. A stepwise moving window search algorithm suitable for selecting features with a fixed order was developed to select in-vehicle noise features. Sound pressure levels at 400, 500, 630 and 800 Hz of in-vehicle noise were fed into the PPNN, and the average accuracy could reach 96.43%. The bogie acceleration characteristics calculated by the multi-body dynamics simulation model were used to recognize normal rail amplitudes of 0.1 and 0.2 mm. The bogie acceleration was decomposed by the complete ensemble empirical mode decomposition with adaptive noise and a reconstructional signal was obtained. The energy entropy of the reconstructional signal was fed into the PPNN, and the average accuracy could reach 95.40%. The urban rail transit network may also affect the service performance of infrastructures. Liu *et al*. [12] proposed passenger flow anomaly detection in an urban rail transit network with GCN-informer and Gaussian Bayes models. They proposed a novel anomaly detection methodology based on a deep-learning framework consisting of a GCN-informer model and a Gaussian Naive Bayes model. The GCN-informer model was used to capture the spatial and temporal features of inbound and outbound passenger flow, and it was trained on normal datasets. The Gaussian Naive Bayes model was used to construct a binary classifier for anomaly detection, and its parameters were estimated by feeding the normal and abnormal test data into the trained GCN-informer model. Experiments were conducted on a real-world URTN passenger flow dataset in Beijing. The results showed a superior performance of the proposed framework compared with existing anomaly detection algorithms in detecting network-level passenger flow anomalies.

We would like to thank the Editor-in-Chief, the Commissioning Editor and all the editorial board members of *Philosophical Transactions A* for their help and support during the production of this theme issue. We would also to thank the reviewers of this theme issue for their valuable comments and suggestions.

## Data Availability

This article has no additional data.
